# Translation of the Shoulder Pain and Disability Index and psychometric evaluation of the Swedish version

**DOI:** 10.1016/j.jseint.2026.101638

**Published:** 2026-01-28

**Authors:** Anna H. Petersson, Jenny M. Samuelsson, Hanna C. Björnsson Hallgren, Johan H. Scheer, Theresa M. Holmgren

**Affiliations:** a Department of Health, Medicine and Caring Sciences, Division of Prevention, Rehabilitation and Community Medicine, Unit of Physiotherapy, Linköping University, Linköping, Sweden; b Department of Orthopaedics, Linköping University Hospital, Linköping, Sweden; c Department of Rehabilitation in Motala, Motala, Sweden; d Division of Orthopaedic Surgery, Department of Biomedical and Clinical Sciences, Linköping University, Linköping, Sweden

**Keywords:** Patient-reported outcome measures, Minimal important change, Subacromial pain, Validation study, Outcome assessment, Responsiveness, Reliability

## Abstract

**Background:**

The Shoulder Pain and Disability Index (SPADI) is a widely used shoulder-specific patient-reported outcome measure. This study aimed to translate and adapt SPADI into Swedish, to evaluate psychometric properties and determine the minimal important change (MIC) in patients with subacromial pain.

**Methods:**

This study was conducted in 2 phases. Phase 1 involved translation and adaptation to Swedish using forward-backward translation and patients' feedback. In phase 2, 158 patients, with shoulder pain and disability due to subacromial pain, were recruited to evaluate validity, reliability, responsiveness and MIC.

**Results:**

There were no disagreements during the translation process, and patients found the questions relevant and comprehensible. The SPADI and 7-item Disabilities of the Arm, Shoulder, and Hand scores showed a high correlation (0,86). Internal consistency with Cronbach's alpha 0,93 (n = 158). Test–retest reliability with Intraclass Correlation Coefficient 0,87, (n = 60). The standard error of measurement was 3,8 and the minimal detectable change was 10,5 in the total score. Responsiveness had an area under the curve of 0,92 (95% confidence interval 0,9 to 1) generating a MIC of 11, with sensitivity at 92% and specificity at 75% (n = 107).

**Conclusion:**

The SPADI was successfully translated and adapted into Swedish and demonstrated excellent psychometric properties in patients with subacromial pain. Additionally, MIC values have been established to identify clinically meaningful changes, useful in both research and clinical practice. These findings support its use in this population, while further research should confirm validity for other shoulder conditions.

Patient-reported outcome measures (PROMs) are utilized to capture patients' perspectives on their health status.^10^ These tools are essential evaluating outcomes in patient care and in medical research. The Shoulder Pain and Disability Index (SPADI)^31^ is a PROM designed to assess pain and disability related to the shoulder and is one of the most widely used self-assessed outcome measures for shoulder research and clinical assessments.^6^^,^^27^^,^^35^ The instrument has been applied extensively in conditions such as frozen shoulder, osteoarthritis and subacromial pain addressing areas where other instruments may not sufficiently cover both pain and function.^20^ The English version of SPADI has been translated into several languages and has demonstrated strong measurement properties across various contexts.^20^ Another crucial measurement property is the minimal important change (MIC), which represents the smallest change in the score that a patient perceives as meaningful.^11^ Despite its importance, the number of studies that have assessed the MIC for SPADI remains limited, indicating the need for further validation and replication in diverse populations and settings. In Sweden, there is a lack of shoulder-specific PROMs with robust measurement properties applicable to the most common diagnosis, subacromial pain. Existing instruments are either condition-specific, not patient-reported, or lack validation and MIC values for this population. Translating and adapting SPADI into Swedish addresses this gap by providing a brief, region-specific tool that combines pain and disability, and supports international comparability in research. Up until now, no validated Swedish translation of SPADI exists.

The purpose of this study was to translate and adapt the English version of SPADI into Swedish, to evaluate the psychometric properties and determine the MIC of the Swedish version in patients with shoulder pain and disability due to subacromial pain.

## Materials and methods

### Study design

**Figure 1:**
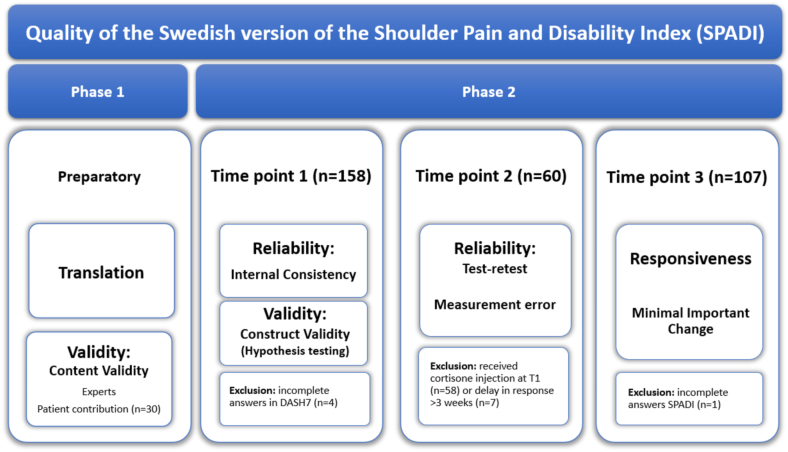
Overview of the target psychometric properties of the SPADI across study phases and time points (T1: baseline, T2: 1 week, T3: 3 months). *SPADI*, Shoulder Pain and Disability Index; *DASH7*, 7-item Disabilities of the Arm, Shoulder, and Hand.

This is a cohort study with prospectively collected data from February 2023 to December 2024, conducted in 2 phases. Phase 1 involved translation and adaptation of the English version of SPADI into Swedish using forward–backward translation and pretesting of the instrument in patients with shoulder pain and disabilities to verify content validity outlined by Beaton et al.^2^ Phase 2 evaluated the psychometric properties of the translated version of SPADI following the Consensus-based Standards for the Selection of health status Measurement Instruments (COSMIN) checklist,^23^ COSMIN guidelines for taxonomy and definitions^24^ and guidelines for reporting.^14^ A detailed overview of the study design and the psychometric properties evaluated in this study is presented in Fig. 1.

### Phase 1: the process of translation and cross-cultural adaptation

Initially, 2 forward translations of the original SPADI were independently conducted by coworkers at the orthopedic department at the Linköping University Hospital in Sweden. They were native in Swedish and fluent in English. The first translator, an upper-extremity surgeon, and the second translator, a physiotherapist, both unfamiliar with the instrument, independently translated the original version. The 2 translators and 2 methodologists (coauthors J.Sa. and T.H.) discussed discrepancies between the translations from a cultural and linguistic perspective, and consensus was reached to create a single version for back translation.

Next, 2 native English-speaking translators, fluent in Swedish unaware of the study's aims, independently back translated the synthesized Swedish version into English. The 2 translators and the 2 methodologists discussed discrepancies between the back translations and consensus was reached into a synthesized English version. This version was compared to the original version and any discrepancies were discussed to ensure that the Swedish SPADI was both comprehensible and culturally relevant.

To assess the comprehensibility and relevance of the SPADI in a cross-cultural context, 30 patients with ongoing shoulder pain and disabilities, due to various shoulder diagnoses, were conveniently recruited at the orthopedic department at the Linköping University Hospital in Sweden. The aim was to ensure a representative sample in terms of age and gender. These patients completed the SPADI and responded to additional survey questions concerning the interpretation of the instructions, the relevance and comprehensiveness of the items, and the comprehensibility of the response options, see Supplementary File 1. This feedback was used to refine the final version of the SPADI by improving its clarity and ensuring its cultural adaptability.

### Phase 2: evaluation of psychometric properties

#### Participants

Patients with long-standing subacromial pain, who were referred to the orthopedic department in Linköping between May 2023 and September 2024, were recruited in phase 2. All patient referrals for shoulder pain were reviewed by an orthopedic surgeon and if there was no trauma, previous injuries or surgeries to the affected shoulder, as well as no osteoarthritis radiographically verified, the patients were sent to a physiotherapist specialized in shoulders for clinical assessment including diagnostic tests and ultrasound. The subacromial pain diagnosis was based on clinical findings, anamnesis and the ultrasound findings including degenerative or inflammatory changes affecting the subacromial structures. Patients diagnosed with subacromial pain were conveniently invited to participate and included in the study if they consented. Insufficient understanding of the Swedish language was an exclusion criterium.

SPADI was administered at the clinic, at time point 1 (T1) along with the 7-item Disabilities of the Arm, Shoulder, and Hand questionnaire (DASH7).^26^ SPADI was then sent out to the participants by mail 1 week later fort test–retest analysis, at time point 2 (T2). Patients were excluded from the test–retest analysis if a response interval exceeded three weeks from T1, or if they received a steroid injection at the inclusion or any other treatment influencing their shoulder status. Three months after inclusion at time point 3 (T3), SPADI was administrated to the participants by mail together with the Patient Global Impression of Change (PGIC).^19^

#### Outcome measures

The SPADI is considered a reflective model capturing the underlying construct of shoulder pain and disability. It consists of 2 unidimensional subscales, where the first scale includes 5 questions estimating “Pain” and the second scale includes 8 questions estimating “Disability.” Grading of “Pain” or “Disability” goes from 0 to 10, where 0 means "no pain or functional impairment" and 10 means "maximum pain or functional impairment." The subscales generate individual subscores and contribute to a total score ranging from 0 to 130. The sum of the scores is divided by the maximum possible score and multiplied by 100 and is used as a percentage score from 0 to 100, where a higher score reflecting a higher level of pain and disability.^31^

The DASH7^26^ is a shortened version of the Disabilities of the Arm, Shoulder, and Hand (DASH), which is a widely used tool for assessing upper-extremity disabilities using a reflective model.^18^ DASH7 is validated for patients with subacromial pain and solely focuses on shoulder-specific activities. DASH7 offers similar effect size (Cohen's d) of 0.93 as compared to the original DASH (0.92).^26^ It also exceeds the effect size of QuickDASH (0.85),^3^ another shortened version consisting of 11 items. DASH7 demonstrates comparable internal consistency to QuickDASH, with Cronbach's alpha values of 0.84. The 7 items are scored on a 5-point Likert scale ranging from 1 (no difficulty) to 5 (unable to perform), with a transformed total score ranging from 0 (no disability) to 100 (most severe disability).^26^

The PGIC scale^19^ is a standardized tool used to assess patients' perception of changes in their condition over time. In this study, a modified version of the PGIC scale^17^ was used to assess patients' perception of changes after a 3-month follow-up (T3) compared to the start of the study (T1). The question worded “Since beginning of treatment, how would you describe the change in pain, disability, emotions, and overall quality of life related to your pain condition of your shoulder?” Answers were rated on a 5-point Likert scale, “Completely recovered,” corresponded to 1, “Large improvement,” to 2 “Small improvement,” to 3 “Unchanged,” to 4 and “Worse,” to 5.

### Statistical analysis

Missing data in the SPADI scores were adjusted by the average score of the answered items within the same subscale.^31^ Responses in the SPADI with more than 2 missing answers at any time point (T1, T2, or T3) was excluded from the analysis. More than 1 missing answer in the DASH7 at T1 were excluded from the analysis. Paired t-tests were conducted to compare scores within groups, while independent t-tests were used to evaluate differences between groups at each time point. A *P* value <.5 was considered significant in statistical testing. All data were analyzed in SPSS, version 26 (IBM Corp., Armonk, NY, USA).

### Validity

Hypothesis testing for construct validity was done using data from participants at T1 by analyzing correlations between DASH7 and SPADI total scores and the subscores, using Spearman's rank correlation coefficients (r_s_). Cut-offs were defined as low correlation between r_s_ = 0,30 to 0,50, moderate correlation r_s_ = 0.50 to 0.69, and high correlation r_s_ ≥ 0.70.^25^ The hypothesis was that there would be a high positive convergent correlation (r_s_ > 0.7) between the translated and cross-cultural adapted SPADI total score and DASH7, as well as between DASH7 and the SPADI disability score. The correlation between DASH7 and the SPADI pain score was expected to be moderate (r_s_ = 0.50 to 0.69), based on prior studies using DASH or QuickDASH.^15^^,^^21^^,^^28^

### Reliability

#### Internal consistency

Internal consistency was analyzed using data reported from participants at T1. Internal consistency was analyzed separately for total score, pain, and disability with Cronbach's alpha. A value between 0.50 and 0.69 was considered poor, 0.70 to 0.79 acceptable, 0.80 to 0.89 good, and >0.90 excellent.^8^^,^^34^

#### Test–retest reliability

The test–retest reliability of SPADI was determined by the Intraclass Correlation Coefficients (ICC_agreement_), using measurements from T1 comparing it to measurements reassessed 1 week later (T2).^22^ The absolute agreement was controlled for in a 2-way mixed effects model^30^ for the total score, for the 2 subscores, and individually for each item 1 to 13 in the questionnaire. The ICC_agreement_ single-measurement scores of <0.40 were considered poor correlation, 0.40 to 0.59 fair, 0.60 to 0.74 good and >0.75 excellent.^7^ A Bland-Altman plot was used to visualize the distribution of mean differences between the 2 measurements T1 and T2 of SPADI pain, disability and total score and with the limits of agreement (LOA). LOA were defined as LOA = $\bar{d}$ ±1.96 x SDd, where $\bar{d}=$ the mean difference of the measurements and SDd = standard deviation (SD) of the difference.^5^

#### Standard error of measurement

The standard error of measurement (SEM_agreement_) was calculated with the formula; SEM=SDx√(1−ICC), where SD was the standard deviation for the mean change of the SPADI total score from T1 to T2 and ICC was the ICC for SPADI total score.^36^

#### Minimal detectable change

Minimal detectable change (MDC) was calculated with the formula MDC=zx√2xSEM, where z = 1.96 was the z score for estimating a 95% confidence interval (CI), √2 signified the 2 time points (T1 and T2) of SPADI measurements and SEM was the standard error of measurement.^4^

### Responsiveness

The responsiveness of the SPADI total change score between T1 and T3 were plotted in a receiver operating characteristic (ROC) curve, with the PGIC serving as the external criterion in an anchor-based approach. The PGIC was dichotomized into “Importantly improved” (“completely recovered”, or “large improvement” in PGIC) or “Not importantly improved” (“small improvement”, “unchanged” or “worse” in PGIC) and this new variable was applied as the anchor. In addition, the PGIC responses were also dichotomized into “No change” (comprising “small improvement' and “unchanged”) and “Worsened” to facilitate the calculation of MIC Deterioration. The ROC area under the curve (ROC AUC) was used to indicate the ability of the SPADI to distinguish between participants who have importantly improved and those who have not, based on the PGIC anchor. A value of 1 signified perfect accuracy, while a value of 0.5 indicated performance no better than chance. Responsiveness was estimated adequate when the AUC was at least 0.7^13^ and the AUC was calculated with a 95% CI.

### Interpretability

#### Minimal important change

MIC was determined using Youden's index, which identifies the optimal cut-off point in the ROC curve. This cut-off maximizes sensitivity (true positive rate) and specificity (true negative rate), ensuring the most accurate distinction between participants who have meaningfully improved and those who have not, based on the PGIC as an anchor.^11^

#### Floor and ceiling effects

Floor and ceiling effects was evaluated at T1 and considered, when more than 15% in the cohort, achieved the lowest or highest possible subscore or total score.^34^

## Results

### Phase 1: translation and cross-cultural adaptation

The forward translation of the SPADI revealed no significant issues or language barriers and after achieving equivalence, the Swedish version of SPADI was fully adapted and validated for the Swedish context, see Supplementary File 2. Back translations illustrated some slight differences but generally reflected the same content as the original version.

The prefinal version was tested in 30 participants (mean age 52 ± 14 years, range 28-84; 57% men) with mean SPADI total score 54 (SD 21). 97% of the participants found the questions relevant to their personal experiences and had no decisive feedback to the items resulting in changes of the instrument. Reported comments, experiences and understanding of the SPADI is presented in Supplementary File 3.

### Phase 2: evaluation of psychometric properties

A total of 158 participants (mean age 58 ± 13 years, 44% men) completed SPADI and DASH7 at time point 1 (T1). Completeness of responses and the distribution of scores (for each item, subscores and total score) at T1 is presented in Supplementary File 4. Sixty participants completed SPADI at time point 2 (T2) and 107 completed SPADI and PGIC at time point 3 (T3) (Fig. 1). The participant demographics and mean values of SPADI at T1, T2, and T3 are shown in Table I.

**Table I:** Participants' demographics.

Characteristic	T1 (n = 158)	T2† (n = 60)	T3 (n = 107)
Sex			
Male	70 (44%)	27 (45%)	43 (40%)
Female	88 (56%)	35 (58%)	59 (55%)
Age (min;max)	58 ± 13 (22;87)	60 ± 13 (27;82)	60 ± 14 (22;87)
Pain duration			
3-6 mo	11 (7%)	7 (12%)	8 (8%)
6 mo-24 mo	92 (58%)	35 (58%)	67 (63%)
>24 mo	55 (35%)	18 (30%)	32 (30%)
SPADI pain, mean (SD)	62 (21)	56 (22)	50 (27)
SPADI disability, mean (SD)	49 (24)	46 (24)	41 (28)
SPADI total score, mean (SD)	54 (22)	50 (22)	45 (27)
DASH7∗	58 (21)	N/A	N/A

*SPADI*, Shoulder Pain and Disability Index; *SD*, standard deviation; *N/A*, not available.

Shoulder Pain and Disability Index and the 7-item Disabilities of the Arm, Shoulder, and Hand questionnaire are presented with mean and standard deviation at time point 1 (T1) (baseline), time point 2 (T2) (1 week), and at time point 3 (T3) (3 mo). 0 represents no pain or functional disability, while 100 signifies the maximum level of pain and disability in both SPADI and DASH7.

∗ n = 154.

† Test–retest.

### Validity

The Spearman's rho for the correlation between SPADI total score and DASH7 score at T1 was 0.86, *P* = <.001. Correlation between SPADI pain score and DASH7 score was 0.72 *P* = <.001 and between SPADI disability score and DASH7 score was 0.86, *P* = <.001. All correlations were considered high.^25^

### Reliability

#### Internal consistency

The internal consistency of the SPADI total score (13 items) showed a Cronbach's alpha coefficient of 0.93, indicating an excellent level of reliability.^15^^,^^21^ The subscale SPADI pain (5 items) had a Cronbach's alpha coefficient of 0.84 and SPADI disability (8 items) a Cronbach's alpha coefficient of 0.91.

#### Test–retest reliability, standard error measurement and minimal detectable change

**Figure 2:**
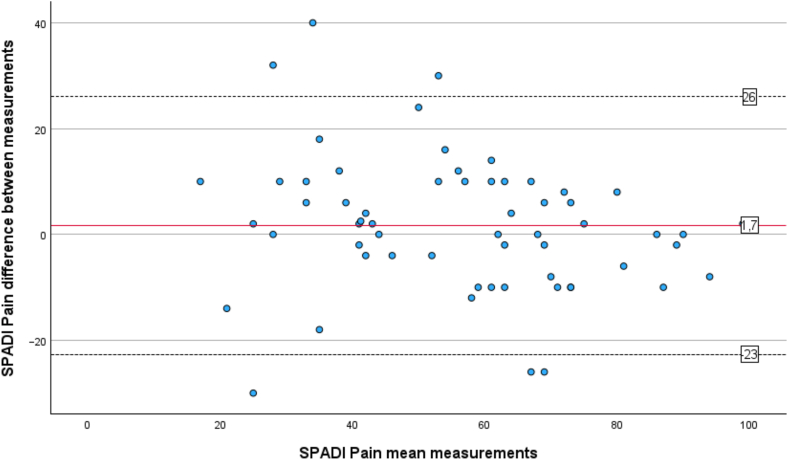
Bland-Altman Plot for the SPADI pain score agreement of test–retest. The mean value of both time points is plotted against the difference between each participant's scores. The mean differences are indicated by the solid line, and the 95% limits of agreement (mean difference ± 1.96 x standard deviation of the difference) are shown by the dashed lines. *SPADI*, Shoulder Pain and Disability Index.

**Figure 3:**
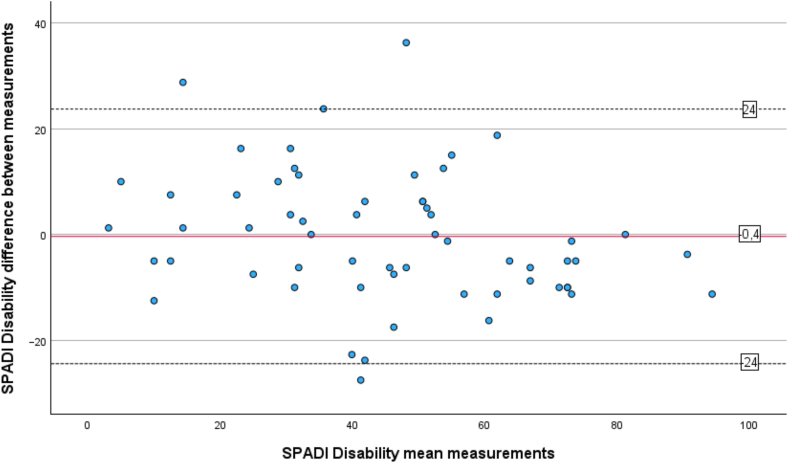
Bland-Altman Plot for the SPADI disability score agreement of test–retest. The mean value of both time points is plotted against the difference between each participant's scores. The mean differences are indicated by the solid line, and the 95% limits of agreement (mean difference ± 1.96 x standard deviation of the difference) are shown by the dashed lines. *SPADI*, Shoulder Pain and Disability Index.

**Figure 4:**
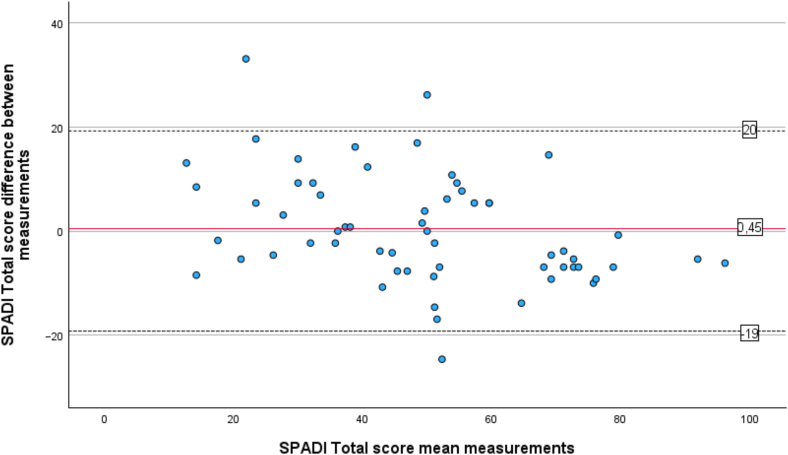
Bland-Altman Plot for the SPADI total score agreement of test–retest. The mean value of both time points is plotted against the difference between each participant's scores. The mean differences are indicated by the solid line, and the 95% limits of agreement (mean difference ± 1.96 x standard deviation of the difference) are shown by the dashed lines. *SPADI*, Shoulder Pain and Disability Index.

A test–retest group of totally 60 participants completed SPADI at T2, (mean age 60 ± 13 years, 45% men), Table I. The mean interval between assessments (T1 and T2) was 10 days (SD 3.4), with no significant differences in SPADI total, pain, or disability scores according to paired-sample t-test. The ICC value for the SPADI total score was 0.87 and indicated an excellent correlation, ICC value for SPADI pain was 0.81 and ICC value for SPADI disabilities was 0.85. ICC values for each item in SPADI ranged between 0.49 to 0.81 and are presented in Table II. The mean differences between the 2 sets of SPADI scores (T1 and T2) fell primarily within the LOA for pain (Fig. 2), disability (Fig. 3), and total score (Fig. 4). SEM was calculated to 3.8 and the MDC was 10.5 in the SPADI total score.

**Table II:** Test–retest reliability for the Shoulder Pain and Disability Index.

Item	ICC∗ (95% CI)
1	0.49 (0.27-0.66)
2	0.81 (0.71-0.88)
3	0.77 (0.64-0.86)
4	0.70 (0.55-0.81)
5	0.70 (0.55-0.81)
Pain subscore	0.81 (0.70-0.88)
6	0.79 (0.67-0.87)
7	0.71 (0.56-0.82)
8	0.72 (0.57-0.83)
9	0.69 (0.52-0.80)
10	0.78 (0.65-0.86)
11	0.79 (0.67-0.87)
12	0.78 (0.65-0.86)
13	0.76 (0.63-0.85)
Disability subscore	0.85 (0.76-0.91)
SPADI total score	0.87 (0.79-0.92)

*CI*, confidence interval; *SPADI*, Shoulder Pain and Disability Index; *ICC*, intraclass correlation coefficient.

∗ Intraclass correlation coefficients values for single-measurement, absolute agreement, 2-way mixed effects model assessed at time point 1 (baseline) and time point 2 (1 week) (n = 60).

## Responsiveness

After 3 months (95 days SD (10), (T3)), 107 out of 158 (68%) participants responded to the SPADI and PGIC questionnaires (mean age 60 ± 14 years, 40% men), Table I. At T3, 36% reported that they were either recovered or had a large improvement, while 55% reported small improvement or no change in PGIC compared to baseline. Additionally, 10 participants (9%) reported that their condition had worsened. Table III presents SPADI total scores at T1 and T3, as well as the mean change between these time points, in relation to the PGIC categories and the dichotomized classification (not importantly improved vs. importantly improved) used in the ROC analysis.

**Table III:** The Shoulder Pain and Disability Index total scores by Patients Global Impression of Change categories and dichotomized groups (n = 107).

Categories of PGIC and dichotomized group	Dichotomized group∗	n	SPADI total score at T1: Mean (SD)	SPADI total score at T3: Mean (SD)	SPADI total score T1-T3: Mean change (SD)
Completely recovered	Importantly improved	2	43 (8)	1 (1)	42 (7)
Large improvement	Importantly improved	36	53 (22)	23 (17)	31 (17)
Importantly improved total		38	53 (21)	22 (17)	31 (17)
Small improvement	Not importantly improved	37	56 (24)	51 (23)	5 (14)
Unchanged	Not importantly improved	22	64 (15)	64 (18)	−0.1 (11)
Worse	Not importantly improved	10	53 (14)	66 (15)	−13 (17)
Not importantly improved total		69	58 (20)	58 (22)	0.8 (15)
*P* value† (95% CI)			*P* = .184 (−14 to 3)	*P* = <.001 (−44 to −28)	*P* = <.001 (24 to 37)

*SPADI*, Shoulder Pain and Disability Index; *CI*, confidence interval; *PGIC*, Patients Global Impression of Change; *SD*, standard deviation.

T1: baseline, T3: 3-mo follow-up.

∗ Dichotomized groups used in the ROC analysis.

† Independent t-test comparing dichotomized groups.

**Figure 5:**
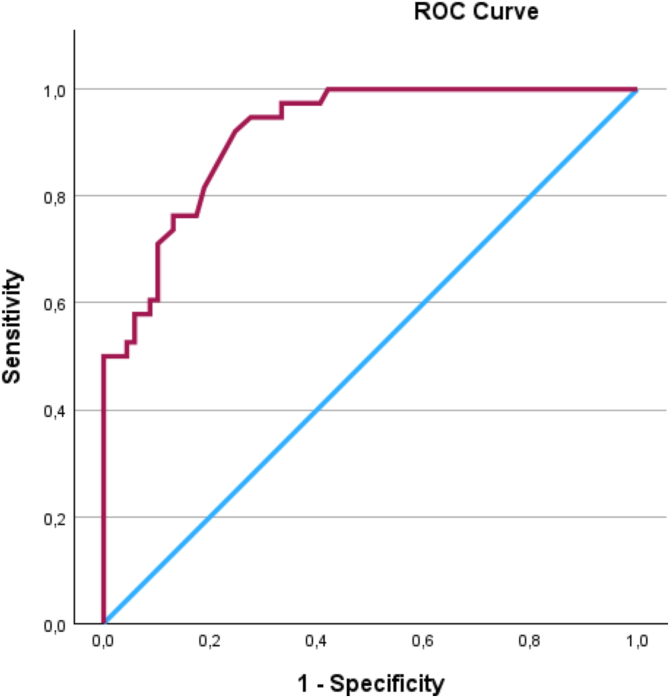
ROC curve showing the ability of different cut-off points in the Shoulder Pain and Disability Index total score change to distinguish between participants who improved meaningfully and those who did not, with corresponding sensitivity and specificity values. *ROC*, receiver operating characteristic.

**Figure 6:**
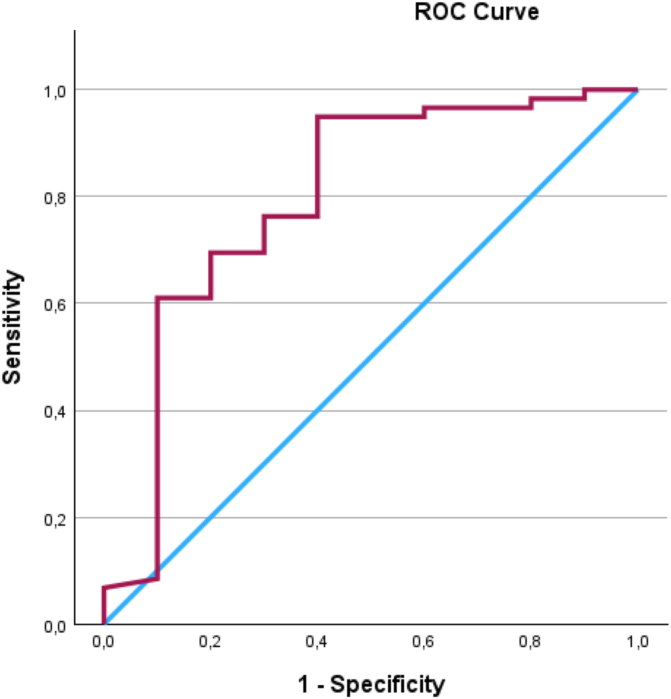
ROC curve showing the ability of different cut-off points in the Shoulder Pain and Disability Index total score change to distinguish between participants who were unchanged and deteriorated, with corresponding sensitivity and specificity values. *ROC*, receiver operating characteristic.

The ROC analysis yielded an AUC of 0.92 (95% CI 0.9 to 1), Fig. 5. This suggests that the SPADI is effective in distinguishing between participants who have experienced clinically meaningful improvements and those who have not and indicate a high level of responsiveness. The ROC AUC for distinguishing between participants with deterioration from “unchanged or not importantly improved” yielded an ROC AUC of 0.8 (95% CI 0.6 to 1), Fig. 6.

## Interpretability

### Minimal Important Change

The threshold value at the ROC curve for MIC was 11, which provided a good balance between sensitivity of 92% and specificity 75%, and had the highest Youden's Index (0.68), resulting in a misclassification rate of 19%. This suggests that an improvement of 11 points or more in the SPADI total score is experienced as a meaningful change by the participant.

For deterioration, the cut-off value in the ROC curve of −15.4 had the highest Youden's Index (0.55), with a sensitivity of 95% and specificity of 60%. A decrease in the total SPADI score by 15 points can thus indicate clinically relevant deterioration for the participant.

It was observed that less than 15% of the participants achieved either the maximum or minimum in subscores or total scores at T1 and therefore, the SPADI demonstrated no ceiling or floor effects, see Supplementary File 4.

## Discussion

The main findings of the study indicate that the SPADI was successfully translated and adapted into Swedish. Its psychometric properties were evaluated, confirming that SPADI is a reliable and robust instrument with good validity and reliability for assessing shoulder pain and disabilities due to subacromial pain in a Swedish context. Additionally, a MIC value was established for both improvement and deterioration, and SPADI has good precision to successfully distinguish between importantly improved and not importantly improved patients.

### Comparison to other studies

Overall, the correlations found in our study for SPADI and DASH7, are consistent with the correlations found in previous studies.^6^^,^^31^^,^^32^ These findings support the construct validity of the Swedish SPADI and its utility in clinical settings for evaluating shoulder pain and disability within the spectrum of subacromial pain. We found no significant floor or ceiling effects which is in accordance with previously studies.^6^^,^^32^

The structural validity of SPADI has been assessed across various studies, supporting a 2-factor structure consisting of pain and disability subscales. However, discrepancies such as cross-loading of items between these subscales highlight limitations in structural validity and the need for cautious interpretation.^20^ However, we did not do a separate factor analysis; instead, we relied on previously published evidence supporting the 2-factor structure of the SPADI. The decision to adopt a 2-factor model remains widely accepted due to its clinical and practical relevance and the internal consistency, as measured by Cronbach's alpha, typically exceeds 0.9, indicating high reliability.^16^^,^^32^ Our study corroborates these results, with Cronbach's alpha coefficient 0.93 for the SPADI total score, 0.84 for the pain score, and 0.91 for the disability score, all indicating excellent reliability. One potential area for discussion is the relevance of individual items in the questionnaire. A high internal consistency (Cronbach's alpha >0.9) suggests that items are measuring the same underlying construct and might indicate redundancy.^34^ Therefore, it might be worthwhile to evaluate if the questionnaire could be shortened without losing its importance, ensuring all items are relevant for the target population. Similar considerations were discussed by Roy et al,^32^ where the reliability and validity of various shoulder function questionnaires, including SPADI, were evaluated.

Originally, SPADI's reproducibility was considered suboptimal, with an ICC of 0.66.^31^ However, systematic reviews have since reported higher reliability coefficients, with ICC values ranging from 0.84 to 0.95.^1^^,^^32^ Our study supports these findings, demonstrating an ICC value of 0.87 for the total score, alongside strong consistency in pain (ICC = 0.81) and disability scores (ICC = 0.85). Higher ICC values observed in recent studies may be attributed to improvements in patient selection, larger and more diverse samples, and refinements in SPADI administration over time, collectively enhancing its reproducibility.

While overall ICC values indicate strong reliability, variability remains in the test–retest measurements of this study. Previous studies reported SEM values between 5 and 7 and MDC between 12 and 20,^20^ serving as an acceptable reference for interpreting our results, as they provide a comparative framework for evaluating the precision of SPADI scores. Differences in SEM values across studies may stem from variations in calculation methods and population characteristics. Additionally, fluctuations between assessments points suggest that SPADI measurements may not be entirely stable, highlighting the need for careful evaluation in clinical applications.

SPADI has been established as a responsive instrument for detecting changes in shoulder pain and disability over time across various shoulder diagnoses,^6^^,^^9^ and our findings further confirm its responsiveness in patients with subacromial pain.

Our study reported an AUC of 0.92, demonstrating a high level of responsiveness and effectiveness in distinguishing clinically meaningful improvements. This result is comparable to Williams et al^37^ reporting an AUC of 0.91.^37^ Furthermore, the ROC curve for MIC threshold of 11 points showed 92% sensitivity, effectively identifying patients with meaningful improvement, and 75% specificity, reducing false positives. However, the 19% misclassification rate indicates that some patients may be incorrectly classified, either as improving when they have not, or as not improving despite experiencing benefits. This reflects the complexity of pain perception and functional recovery, which vary between individuals.

In clinical practice and research, it is crucial to distinguish between statistical significance and clinically meaningful change. The MIC reflects the patient's perspective on what constitutes a meaningful change. For the Swedish SPADI, our findings indicate a MIC of 11 points, meaning that improvements reaching this threshold are considered clinically relevant.^12^ Since the MIC are the same or slightly higher than the MDC of 10.5 points, it ensures that observed changes of 11 are both statistically significant and meaningful for patients. Other studies have reported MIC values ranging from 12 to 20 points,^20^ highlighting variability across populations and study designs. These differences underscore the importance of contextualizing MIC values within specific research settings to accurately assess treatment effects.

### Strengths and limitations

By utilizing well-established guidelines for translation^2^ and evaluation of psychometric properties,^23^ we ensured a rigorous process. Incorporating patient feedback enhanced the relevance and applicability of the SPADI, supporting its content validity. This process ensured that the PROM was comprehensible and relevant to the target population, representing various shoulder diagnosis, ages, and genders. However, several methodological limitations remain: we did not perform a control for different literacy levels, and we did not perform any subgroup analyses to ensure cross-cultural adaptation.

With 158 patients included in this study, the sample size for construct validation was robust,^23^ enhancing the reliability and generalizability of the findings, though the test–retest sample size (n = 60) was lower but sufficient per Terwee et al.^20^ Responsiveness calculations (n = 107) were adequate, yet caution is needed when interpreting the ROC analysis for deterioration due to the small sample (n = 10).

Construct validity could have been further strengthened by including testing of structural validity and by testing multiple hypotheses with different PROMs and assessing both divergent and convergent correlations. The DASH7, a shoulder-specific version of the DASH questionnaire, was selected due to its strong psychometric properties and relevance to assessing shoulder pain and dysfunction in patients with subacromial pain. Its specificity support assessing shoulder-related disabilities without reduction from broader measures approaching the entire arm and hand. However, its limited use in literature and testing exclusively on subacromial pain restricts generalizability.

According to COSMIN guidelines, a 1-3 week interval between assessments is recommended to minimize recall bias and reduce the risk of clinical change.^29^ Our mean interval was 10 days (SD 3), which aligns with these recommendations. However, ensuring individual stability remains challenging, as shoulder symptoms fluctuate with daily activities and pain variability. We attempted to minimize this by including a cohort where 88% had symptoms >6 months and excluding participants who received cortisone injections. Paired-sample t-test confirmed stability at the group level, with no significant differences between T1 and T2 scores. Still, some participants reported changes, which may have influenced test–retest reliability. Practical factors such as different retest environments and varying stressors may also have affected responses, highlighting the inherent difficulty of achieving complete stability in clinical populations. To enhance precision, established statistical methods were used to evaluate SPADI responsiveness, with the PGIC scale providing additional insights into patient-perceived changes. However, the use of PGIC as an external criterion may introduce bias due to its subjective nature. Moreover, although PGIC is widely used, it lacks formal validation in Swedish, as existing translations have not undergone psychometric validation. Schmitt and Abbott^33^ criticized the PGIC, noting that it does not always accurately reflect functional changes over time, with potential recall bias impacting its reliability.^33^ By dichotomizing PGIC responses into simple categories might also lead to the loss of nuanced information about patient improvements but more importantly clinicians need to assess meaningful recovery. While useful for clarity, this categorization should be applied cautiously to avoid overlooking relevant patient experiences. Additionally, de Vet et al^11^ discussed how MIC values depend more on baseline values than on the type of intervention, suggesting that SPADI scores interpretation should consider the initial severity of shoulder conditions.

### Clinical implications

Our results are clinical relevant as SPADI can facilitate standardized assessment and inform treatment planning for a broad range of patients. Furthermore, the calculation of MIC values enhances the interpretability of SPADI scores, enabling clinicians to determine whether observed changes are clinically meaningful for individual patients and to set realistic treatment goals. Validated measurement properties and MIC values strengthens the potential for SPADI to be used as an outcome measure in clinical research, supporting robust evaluation of interventions and comparability across studies. The findings highlight the need for further research to examine the applicability, responsiveness and interpretability of SPADI in other clinical settings and among different shoulder diagnoses in Sweden.

## Conclusion

The SPADI was successfully translated and adapted into Swedish and demonstrated excellent psychometric properties in patients with subacromial pain. Additionally, MIC values have been established to identify clinically meaningful changes, useful in both research and clinical practice. These findings support its use in this population, while further research should confirm validity for other shoulder conditions.
