# Supplementary material for: Translation of the Shoulder Pain and Disability Index and psychometric evaluation of the Swedish version
Source: JSES Int. 2026 Jan 28;10(3):101638. doi: 10.1016/j.jseint.2026.101638 (PMC12972979; doi:10.1016/j.jseint.2026.101638)
Supplement: Supplementary File 1 [file mmc1.docx]

**Supplementary File 1.** Patient survey on the relevance and comprehensibility of the Shoulder Pain and Disability Index

1. Do you think the questions were relevant in describing your shoulder problems?

o Yes, all questions were relevant

o Yes, most of the questions were relevant

o Well, about half of the questions were relevant

o No, only a few questions were relevant

o No, none of the questions were relevant

1. If you think that any of the questions were not relevant, please mark which question(s):

| o Question 1  o Question 2  o Question 3  o Question 4  o Question 5  o Question 6  o Question 7 | o Question 8  o Question 9  o Question 10  o Question 11  o Question 12  o Question 13 |
| --- | --- |

Please describe why you think the questions were not relevant for you:

1. Were the questions easy to understand?

o Yes, all questions were easy to understand

o Yes, most of the questions were easy to understand

o Well, about half of the questions were easy to understand

o No, only a few questions were easy to understand

o No, none of the questions were easy to understand

1. If you think that any of the questions were difficult to understand, please mark which question(s):

| o Question 1  o Question 2  o Question 3  o Question 4  o Question 5  o Question 6  o Question 7 | o Question 8  o Question 9  o Question 10  o Question 11  o Question 12  o Question 13 |
| --- | --- |

1. Please describe why you think these questions were difficult to understand:
2. Please describe if there were any other difficulties in answering the questionnaire:
3. How long would you estimate it took to answer the questionnaire?

o Less than 5 minutes

o 5–10 minutes

o More than 10 minutes
