# Supplementary material for: Translation of the Shoulder Pain and Disability Index and psychometric evaluation of the Swedish version
Source: JSES Int. 2026 Jan 28;10(3):101638. doi: 10.1016/j.jseint.2026.101638 (PMC12972979; doi:10.1016/j.jseint.2026.101638)
Supplement: Supplementary File 2 [file mmc2.docx]

**Supplementary File 2.** Swedish version of The Shoulder Pain and Disability Index

Shoulder Pain and Disability Index (SPADI)

(Smärta och funktionsbegränsning i skuldran)

**Ringa in den siffra som bäst beskriver din smärta/värk i skuldran under den senaste veckan.**

Smärtskala

**Hur mycket besväras du av din smärta/värk?**

Ringa in den siffra som bäst beskriver din smärta/värk där: 0= ingen smärta/värk och 10= värsta tänkbara smärta/värk.

| När det är som värst? | 0 | 1 | 2 | 3 | 4 | 5 | 6 | 7 | 8 | 9 | 10 |
| --- | --- | --- | --- | --- | --- | --- | --- | --- | --- | --- | --- |
| När du ligger på den aktuella sidan? | 0 | 1 | 2 | 3 | 4 | 5 | 6 | 7 | 8 | 9 | 10 |
| När du sträcker dig efter något på en hög hylla? | 0 | 1 | 2 | 3 | 4 | 5 | 6 | 7 | 8 | 9 | 10 |
| När du nuddar nacken? | 0 | 1 | 2 | 3 | 4 | 5 | 6 | 7 | 8 | 9 | 10 |
| När du skjuter ifrån med den aktuella armen? | 0 | 1 | 2 | 3 | 4 | 5 | 6 | 7 | 8 | 9 | 10 |

Skala för funktionsbegränsning

**Hur stora svårigheter har du?**

Ringa in den siffra som bäst beskriver din upplevelse där: 0= inga svårigheter och 10= så svårt att det behövs hjälp.

| Att tvätta håret? | 0 | 1 | 2 | 3 | 4 | 5 | 6 | 7 | 8 | 9 | 10 |
| --- | --- | --- | --- | --- | --- | --- | --- | --- | --- | --- | --- |
| Att tvätta ryggen? | 0 | 1 | 2 | 3 | 4 | 5 | 6 | 7 | 8 | 9 | 10 |
| Att ta på en tröja? | 0 | 1 | 2 | 3 | 4 | 5 | 6 | 7 | 8 | 9 | 10 |
| Att ta på en skjorta med knappar? | 0 | 1 | 2 | 3 | 4 | 5 | 6 | 7 | 8 | 9 | 10 |
| Att ta på dig dina byxor? | 0 | 1 | 2 | 3 | 4 | 5 | 6 | 7 | 8 | 9 | 10 |
| Att placera något på en hög hylla? | 0 | 1 | 2 | 3 | 4 | 5 | 6 | 7 | 8 | 9 | 10 |
| Att bära ett tungt föremål som väger ca 5 kg? | 0 | 1 | 2 | 3 | 4 | 5 | 6 | 7 | 8 | 9 | 10 |
| Att ta ut något från din bakficka? | 0 | 1 | 2 | 3 | 4 | 5 | 6 | 7 | 8 | 9 | 10 |
