# Supplementary material for: Translation of the Shoulder Pain and Disability Index and psychometric evaluation of the Swedish version
Source: JSES Int. 2026 Jan 28;10(3):101638. doi: 10.1016/j.jseint.2026.101638 (PMC12972979; doi:10.1016/j.jseint.2026.101638)
Supplement: Supplementary File 3 [file mmc3.docx]

|  | |
| --- | --- |
| **General relevance** | Most participants found questions relevant to their personal experiences and had no feedback on the items. |
| Compensation and behaviour changes over time | Over time, individuals learn to compensate for their pain or disability, reducing the perceived impact on daily activities. For example, 1 comment highlighted the difficulty in answering due to changes in behaviour over time, such as avoiding using a back pocket (item 13). |
| Task relevance | (Items 10, 13), One respondent indicated no problems with the tasks mentioned, therefore finding them irrelevant. |
| Dressing difficulties and item relevance | One participant mentioned that dressing difficulties led to avoiding certain clothing, affecting their answers related to dressing. Another participant found item 9 irrelevant because a shirt was easier to use than putting on a sweater (item 10). |
|  |  |
| **General understanding** | Several comments indicated no difficulties, suggesting the questionnaire is generally well-structured and comprehensible. |
| Assessment scope | A question was raised about whether the assessment applies to one shoulder or both, indicating a need for clearer instructions. |
| Introductory text | A suggestion to adjust the language in the introductory text for better clarity. |
| Specific item understanding | Item 1: "Pain at its worst" and Item 5: "When you push off with the affected arm" were found difficult to understand by one participant each. |
| Question numbering | One participant observed that the absence of numbers on the questions might have led to confusion. It was suggested to add numbers for every item to improve clarity. |
|  |  |
| **Time to complete the questionnaire** | 19 participants (63%) < 5 min, eight participants (27%) 5-10 min, two participants (7%) > 10 min |
| *Note:* Assessed in the pretesting phase (n=30) | |

**Supplementary File 3.** Participant reported comments, experiences, and understanding of the Shoulder Pain and Disability Index (SPADI)
