# Supplementary material for: Translation of the Shoulder Pain and Disability Index and psychometric evaluation of the Swedish version
Source: JSES Int. 2026 Jan 28;10(3):101638. doi: 10.1016/j.jseint.2026.101638 (PMC12972979; doi:10.1016/j.jseint.2026.101638)
Supplement: Supplementary File 4 [file mmc4.docx]

|  | | | | | | |
| --- | --- | --- | --- | --- | --- | --- |
| **Item** | **Wording of the original SPADI** | **Minimum (%)** | | **Maximum (%)** | **Mean total score (SD)** | **Missing answers** |
| **1** | How severe is your pain at its worst? | 0 (1) | 10 (18) | | 8 (2) | 2 |
| **2** | when lying on the affected shoulder? | 0 (4) | 10 (18) | | 7 (3) |  |
| **3** | when you reach for something on a high shelf? | 0 (3) | 10 (21) | | 7 (3) |  |
| **4** | when you touch the back of your neck? | 0 (18) | 10 (5) | | 4 (3) | 2 |
| **5** | when you push with the involved arm? | 0 (4) | 10 (11) | | 6 (3) | 1 |
|  | **Pain sub score (0-50)** | 0 (0)* | 50 (1)* | | 62 (21) |  |
|  | How much difficulty do you have: |  |  | |  |  |
| **6** | washing your hair? | 0 (17) | 10 (4) | | 5 (3) | 1 |
| **7** | washing your back? | 0 (3) | 10 (23) | | 7 (3) | 1 |
| **8** | putting on an undershirt or pullover sweater? | 0 (8) | 10 (6) | | 5 (3) | 1 |
| **9** | putting on a shirt that buttons down the front? | 0 (20) | 10 (2) | | 4 (3) | 2 |
| **10** | putting on your pants? | 0 (27) | 10 (2) | | 3 (3) |  |
| **11** | placing an object on a high shelf? | 0 (5) | 10 (20) | | 7 (3) | 1 |
| **12** | carrying a heavy object of 10 pounds (4.5 kilograms) at your side? | 0 (17) | 10 (13) | | 5 (3) |  |
| **13** | removing something from your back pocket? | 0 (18) | 10 (7) | | 4 (3) |  |
|  | **Disability sub score (0-80)** | 0 (1)^a^ | 80 (0)^a^ | | 49 (24) |  |
|  | **SPADI total score (0-130)** | 0 (0)^a^ | 130 (0)^a^ | | 54 (22) |  |
| Note: SPADI with achieved minimum or maximum item scores, sub scores and total score presented in %, mean scores with standard deviation (SD) and missing answers. ^a^Floor and ceiling effects were considered present when more than 15% of participants achieved the maximum or minimum possible sub score (pain; 0-50, disability; 0-80) or total score (0-130) | | | | | | |

**Supplementary File 4.** Shoulder Pain and Disability Index (SPADI), item- and total scores at baseline (T1)
